# Vimentin deficiency in macrophages induces increased oxidative stress and vascular inflammation but attenuates atherosclerosis in mice

**DOI:** 10.1038/s41598-018-34659-2

**Published:** 2018-11-19

**Authors:** Liliana Håversen, Jeanna Perman Sundelin, Adil Mardinoglu, Mikael Rutberg, Marcus Ståhlman, Ulrika Wilhelmsson, Lillemor Mattsson Hultén, Milos Pekny, Per Fogelstrand, Jacob Fog Bentzon, Malin Levin, Jan Borén

**Affiliations:** 1 Department of Molecular and Clinical Medicine/Wallenberg Laboratory, University of Gothenburg, and Sahlgrenska University Hospital, Gothenburg, Sweden; 2 Strategic planning and operations, Cardiovascular and metabolic diseases, IMED Biotech Unit, AstraZeneca, Gothenburg, Sweden; 3 Science for Life Laboratory, KTH - Royal Institute of Technology, Stockholm, Sweden; 4 Centre for Host–Microbiome Interactions, Dental Institute, King’s College London, London, SE1 9RT United Kingdom; 5 Department of Clinical Neuroscience/Center for Brain Repair, University of Gothenburg, Gothenburg, Sweden; 6 Department of Clinical Medicine, Aarhus University, Aarhus, Denmark, and Centro Nacional de Investigaciones Cardiovasculares Carlos III (CNIC), Madrid, Spain

## Abstract

The aim was to clarify the role of vimentin, an intermediate filament protein abundantly expressed in activated macrophages and foam cells, in macrophages during atherogenesis. Global gene expression, lipid uptake, ROS, and inflammation were analyzed in bone-marrow derived macrophages from vimentin-deficient (*Vim*^−/−^) and wild-type (*Vim*^+/+^) mice. Atherosclerosis was induced in *Ldlr*^−/−^ mice transplanted with *Vim*^−/−^ and *Vim*^+/+^ bone marrow, and in *Vim*^−/−^ and *Vim*^+/+^ mice injected with a PCSK9 gain-of-function virus. The mice were fed an atherogenic diet for 12–15 weeks. We observed impaired uptake of native LDL but increased uptake of oxLDL in *Vim*^−/−^ macrophages. FACS analysis revealed increased surface expression of the scavenger receptor CD36 on *Vim*^−/−^ macrophages. *Vim*^−/−^ macrophages also displayed increased markers of oxidative stress, activity of the transcription factor NF-κB, secretion of proinflammatory cytokines and GLUT1-mediated glucose uptake. *Vim*^−/−^ mice displayed decreased atherogenesis despite increased vascular inflammation and increased CD36 expression on macrophages in two mouse models of atherosclerosis. We demonstrate that vimentin has a strong suppressive effect on oxidative stress and that *Vim*^−/−^ mice display increased vascular inflammation with increased CD36 expression on macrophages despite decreased subendothelial lipid accumulation. Thus, vimentin has a key role in regulating inflammation in macrophages during atherogenesis.

## Introduction

Intermediate filaments are cytoskeletal and nucleoskeletal structures that contribute to subcellular and tissue-specific biological functions. Vimentin is an abundant intermediate filament protein that is expressed in a variety of cells including fibroblasts, astrocytes, endothelial cells and macrophages^1,2^. Vimentin is important for stress responses of cells and tissues and cellular functions such as cell motility, migration and endocytosis^3,4^. A number of studies suggested a role for vimentin in the control of cell differentiation of various cell types^2,5^. Interestingly, vimentin displays a dramatic increase in expression (94-fold increase) when macrophages engulf atherogenic lipoproteins and become ‘foam cells’^6^. The macrophage foam cells play a critical role in the occurrence and development of atherosclerosis, but the role of vimentin in this process is still unclear.

Atherosclerosis is initiated and driven by the subendothelial accumulation of atherogenic lipoproteins^7–10^. The retained lipoproteins initiate an inflammatory process in the arterial wall that accelerates further accumulation of atherogenic lipoproteins^8^. Interestingly, vimentin has been linked to the innate immunity and shown to regulate activation of the NACHT, LRR and PYD domains-containing protein 3 (NLRP3) inflammasome, a macromolecular complex that orchestrates early inflammatory responses of the innate immune system^11^. In line, decreased active caspase-1 and IL-1β levels have been reported in *Vim*-deficient and vimentin-knockdown macrophages^11^. The NLRP3 inflammasome is activated in response to a broad spectrum of infectious agents^12^. Despite this, Vim-deficient phagocytes have been shown to display increased capacity to mediate bacterial killing by abundant production of reactive oxygen species (ROS) and nitric oxides^13^. Results suggest that vimentin suppresses ROS production by interaction with the p47phox active subunit of the NADPH oxidase^13–15^. Thus, lack of vimentin leads to augmented production of ROS in both mouse and human macrophages^13^, resulting in oxidative damage^16^. ROS has been shown to promote pro-inflammatory signalling in macrophages^17–19^, and linked to endothelial dysfunction^20^. In line, vimentin deficiency results in decreased endothelial relaxation^21^.

Since ROS expression and innate immune responses of macrophages are critical components in atherogenesis^22,23^, we elucidated the role of vimentin for atherogenic response of macrophages. Here we demonstrate that vimentin has a strong suppressive effect on ROS and that vimentin deficiency in macrophages induces impaired endocytosis, and an inflammatory response including increased GLUT1-mediated glucose uptake. We also linked increased surface expression of CD36 to the inflammation. These findings indicate that vimentin has a key role in regulating inflammation in macrophages during atherogenesis.

## Results

### Decreased Lipid Uptake of Native LDL but increased uptake of oxLDL in *Vim*^−/−^ Macrophages

**Figure 1:**
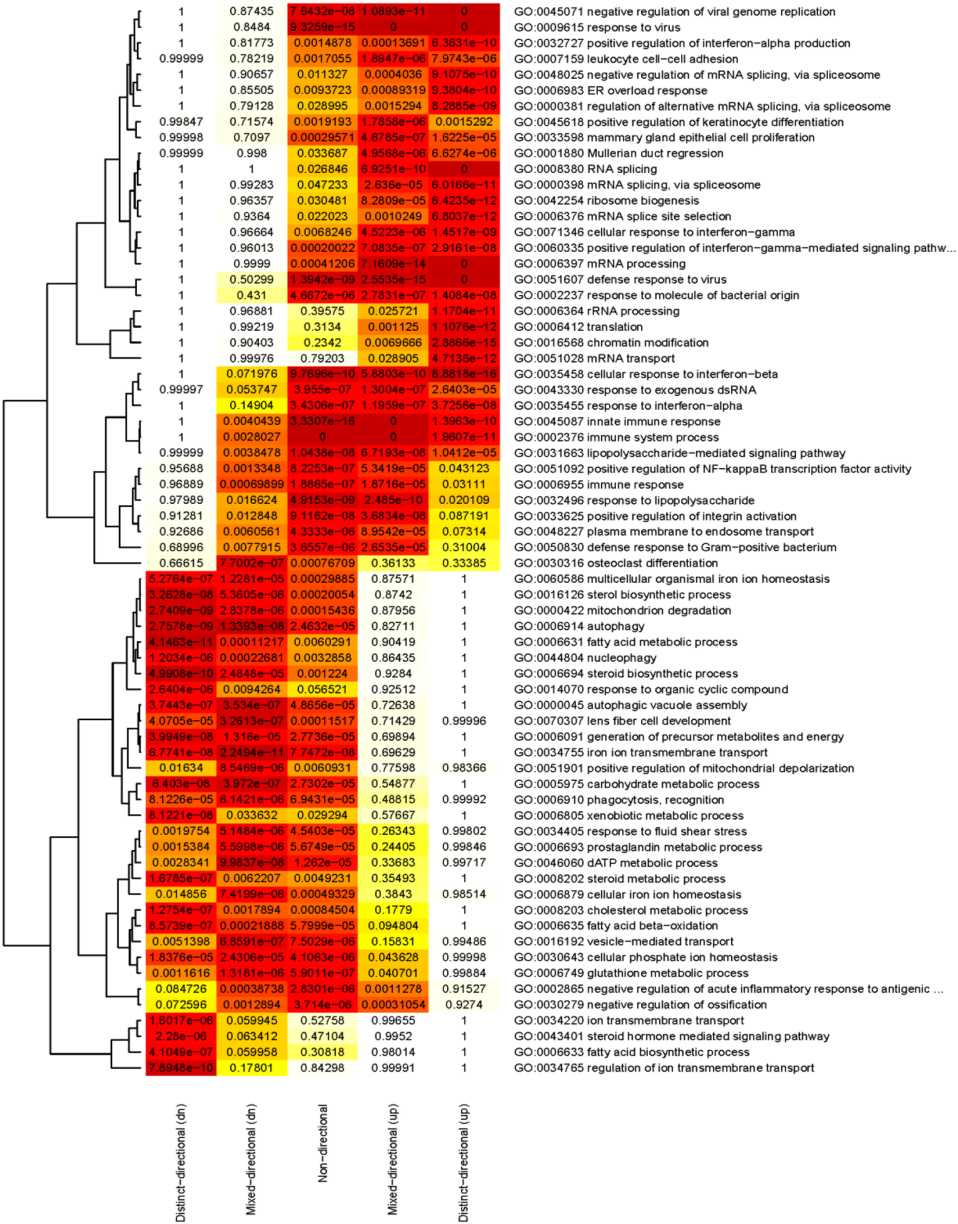
Heatmap shows the significantly changed gene sets and their association to up and downregulated genes based on gene expression data from *Vim*^−/−^ and wild-type macrophages. Gene sets clustered at the upper part of the figure show patterns of mostly upregulated genes whereas the genes sets in the lower part show patterns of mostly downregulated genes. *−*Log (p values) are used for color coding.

To elucidate the consequences of vimentin deficiency in macrophages, we first performed global gene expression profiling of bone marrow–derived macrophages from *Vim*^−/−^ and wild-type mice. We carried out gene set analysis for Gene Ontology (GO) biological process terms and demonstrated that the gene sets with the highest number of significantly vimentin-regulated genes were associated with functions in the immune system process, regulation of NFκB transcription factor activity, defence response to virus, plasma membrane to endosome transport and glutathione metabolic process (Supplementary Fig. S1). To improve the biological interpretation of these results, we also assessed the extent of up or downregulation of genes within the vimentin-regulated gene sets. We found that immune-related biological functions were associated with upregulated genes whereas glutathione metabolic process was associated with downregulated genes (Fig. 1).

**Figure 2:**
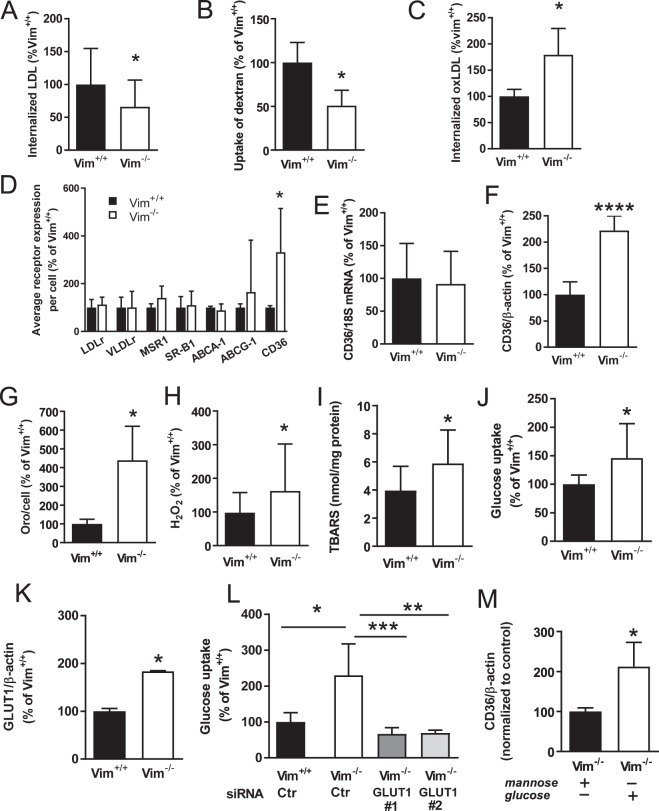
Reduced uptake of native LDL, but increased uptake of oxLDL and protein levels of CD36 in *Vim*^−/−^ bone marrow–derived macrophages. (**A**) Uptake of LDL in bone marrow–derived macrophages from *Vim*^−/−^ and wild-type (*Vim*^+/+^) mice following 3 h incubation with fluorescently labelled native LDL (10 µg/ml). (**B**) Uptake of dextran in bone marrow–derived macrophages from *Vim*^−/−^ and wild-type mice. Cells were incubated for 16 h with 200 µg/ml of Alexa fluor 488 labelled dextran. (**C**) Uptake of oxLDL in bone marrow–derived macrophages from *Vim*^−/−^ and wild-type mice following 3 h incubation with fluorescently labelled oxLDL (10 µg/ml) in the presence of 180 µM oleic acid. (**D**) Surface expression of receptors involved in lipoprotein uptake and cholesterol efflux in bone marrow–derived macrophages from *Vim*^−/−^ and wild-type mice as analyzed by flow cytometry. Macrophage scavenger receptor 1 (MSR1), very low-density lipoprotein receptor (VLDLr), low-density lipoprotein receptor (LDLr), ATP-binding cassette sub-family G member 1 (ABCG1), ATP-binding cassette transporter (ABCA1), scavenger receptor B1 (SR-B1) and cluster of differentiation 36 (CD36). (**E**) CD36 mRNA expression detected by Q-PCR and (**F**). CD36 protein levels detected by immunoblotting in bone marrow–derived macrophages from *Vim*^−/−^ and wild-type mice. (**G**) Lipid accumulation assessed by Oil Red O (OrO) staining in bone marrow–derived macrophages from *Vim*^−/−^ and wild-type mice incubated for 16 h with 180 μM oleic acid (OA). (**H**) Hydrogen peroxide (H_2_O_2_) and (**I**). Lipid peroxidation (TBARS) in bone marrow–derived macrophages from *Vim*^−/−^ and wild-type mice. (**J**) Glucose uptake and (**K**). GLUT1 protein levels in bone marrow–derived macrophages from *Vim*^−/−^ and wild-type mice. (**L**) Glucose uptake in bone marrow–derived *Vim*^−/−^ and wild-type macrophages transfected with control or GLUT1 siRNA. (**M**) CD36 protein levels of bone marrow–derived *Vim*^−/−^ macrophages treated with 6 mg/ml glucose or mannose. Results are shown as mean ± SD, *n* = 18–20 for (**A**,**H** and **I**), *n* = 10−12 for (**B**,**G** and **J**), *n* = 4−8 for (**C–F**,**K**,**L** and **M**). *p < 0.05, **p < 0.01, ****p < 0.0001, Unpaired two tailed t-test in (**A–D**,**F–K**,**M**) *vs Vim*^+/+^ macrophages; One-way ANOVA followed by Sidak’s multiple comparison test (CTR  siRNA transfected Vim^−/−^ macrophages *vs* CTR  siRNA transfected Vim^+/+^ macrophages, and GLUT1 siRNA#1 and GLUT1 siRNA#2 respectively *vs*  CTR siRNA transfected Vim^−/−^ macrophages) in L.

The finding that the global gene expression analysis indicated that the plasma membrane to endosome transport was affected in *Vim*^−/−^ macrophages was interesting since endocytosis of atherogenic lipoproteins is critical for atherogenesis. We therefore analyzed the uptake of LDL in bone marrow–derived macrophages from *Vim*^−/−^ and wild-type mice and found decreased internalization of fluorescently labelled native LDL in *Vim*^−/−^ macrophages (Fig. 2A). Since the uptake of native LDL is mediated mainly by macropinocytosis in macrophages^24^, we then analyzed the uptake of fluorescent dextran as a measure of this endocytic process. We showed that dextran accumulation was significantly decreased in *Vim*^−/−^ macrophages compared with wild-type macrophages (Fig. 2B). In contrast, the uptake of fluorescently labelled mildly oxidized LDL (oxLDL) was higher in *Vim*^−/−^ macrophages than in wild-type macrophages (Fig. 2C). Together, these results show that vimentin deficiency induces a decreased uptake of native LDL, but increased uptake of oxLDL.

### Increased Surface Expression of CD36 on *Vim*^−/−^ Macrophages

We next used flow cytometry to analyse the surface expression of key regulators of lipid metabolism on bone marrow–derived macrophages from *Vim*^−/−^ and wild-type mice. No differences were seen for low-density lipoprotein receptor (Ldlr), very low- density lipoprotein receptor (Ldlr), macrophage scavenger receptor 1 (MSR1), scavenger receptor B1 (SR-B1), ATP binding cassette A 1 or G1 (ABCA1, ABCG1) (Fig. 2D). However, the analysis showed an increased surface expression of the scavenger receptor CD36 on *Vim*^−/−^ macrophages (Fig. 2D). Although CD36 mRNA expression did not differ between bone marrow–derived macrophages from *Vim*^−/−^ and wild-type mice (Fig. 2E), increased CD36 protein levels in *Vim*^−/−^ macrophages were verified by immunoblot (Fig. 2F). Notably, CD36 is one of the principal receptors for oxLDL and using CD36 knockdown with siRNA in wild-type macrophages we confirmed that mildly oxLDL is internalized via this receptor (Supplementary Fig. S2B). Our results showed that the increase in oxLDL uptake correlated with increased surface expression of CD36 on *Vim*^−/−^ macrophages.

Notably, CD36 binds also a number of other ligands including long-chain fatty acids^25^. We therefore incubated *Vim*^−/−^ and wild-type macrophages with 180 μM oleic acid (OA) for 24 hours^25^. Quantification of the total Oil Red O-stained surface area showed significantly higher fatty acid uptake in *Vim*^−/−^ macrophages than in wild-type macrophages (Fig. 2G), thus confirming that the CD36 was functional. These results show that vimentin deficiency promotes increased surface expression of functional CD36 on bone marrow–derived macrophages.

### Increased Oxidative Stress and Glucose Uptake in *Vim*^−/−^ Macrophages Associates with Increased Surface Expression of CD36

Lack of vimentin has been shown to augment production of ROS in macrophages^13^. We therefore confirmed these results by analysing levels of hydrogen peroxide and a byproduct of lipid peroxidation, thiobarbituric acid-reactive substances (TBARS) and found that these were significantly higher in *Vim*^−/−^ macrophages than in wild-type macrophages (Fig. 2H,I). In line, the global gene expression analysis indicated downregulated genes linked to glutathione metabolism (*i.e*., protection against oxidative stress). We confirmed decreased glutathione reductase expression in bone marrow–derived *Vim*^−/−^ macrophages using RT-PCR (Supplementary Fig. S3). These results confirm that vimentin deficiency in macrophages leads to increased oxidative stress.

ROS stimulates glucose uptake^26,27^. We therefore analyzed glucose cellular uptake and showed that glucose uptake was significantly higher in *Vim*^−/−^ macrophages than in wild-type macrophages (Fig. 2J). Furthermore, immunoblot analysis of *Vim*^−/−^ and wild-type macrophages showed increased levels of GLUT1, the primary rate-limiting glucose transporter on proinflammatory-polarized macrophages^28^ in *Vim*^−/−^ macrophages (Fig. 2K). We also showed that the increase in glucose uptake in *Vim*^−/−^ macrophages compared with wild-type macrophages was abolished by knockdown of GLUT1 with two different siRNA (Fig. 2L). The GLUT1 protein expression levels after GLUT1 knockdown are shown in Supplementary Fig S4. Interestingly, glucose has been reported to regulate CD36 expression at the translational level in macrophages^29^, we therefore tested if CD36 protein levels could be further increased in *Vim*^−/−^ macrophages by increasing the glucose concentration from 2.5 to 6 mg/ml (Fig. 2M), showing the importance of glucose availability for CD36 expression. No increased in CD36 protein expression was detected in wild-type macrophages by increasing the glucose concentration (Supplementary Fig. S5). These data indicate that vimentin deficiency induces increased expression of GLUT1 and increased glucose uptake, accompanied by increased expression of CD36 on macrophages.

### Increased Inflammation in *Vim*^−/−^ Macrophages is linked to CD36

**Figure 3:**
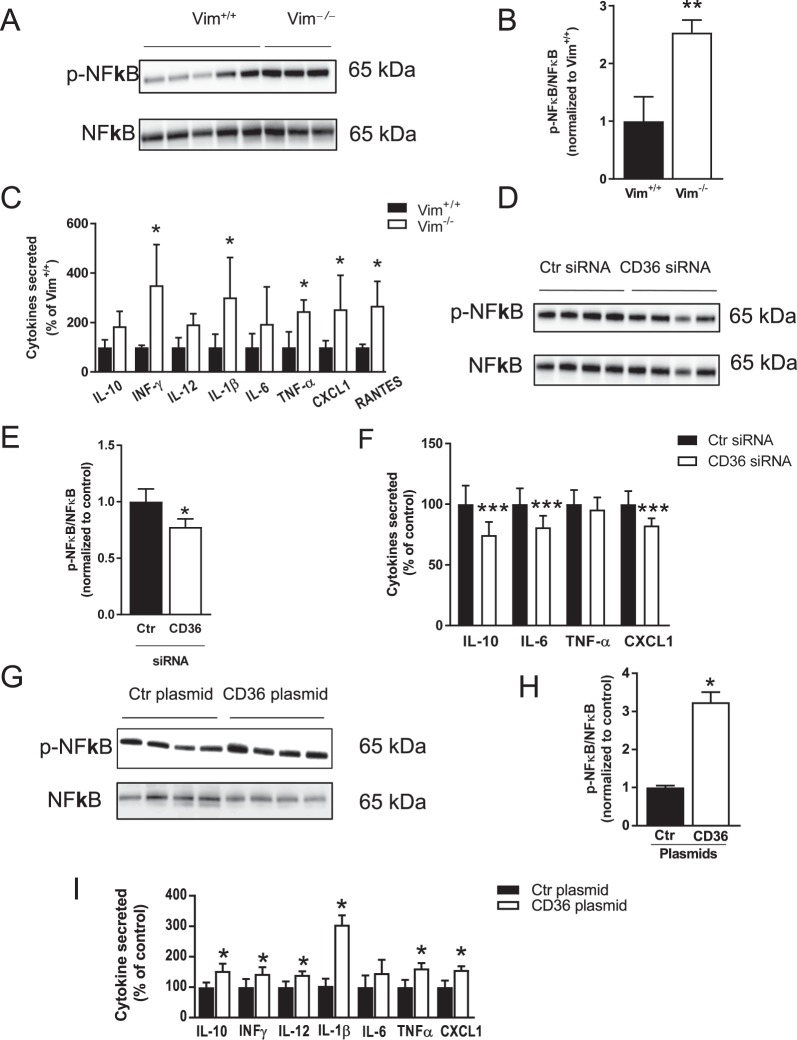
The proinflammatory profile of *Vim*^−/−^ macrophages is linked to CD36. (**A**,**B**) NFκB activation and (**C**). Cytokine secretion in bone marrow–derived macrophages from *Vim*^−/−^ and wild-type mice. (**D**,**E**) NFκB activation and. (**F**) Cytokine secretion in in bone marrow–derived *Vim*^−/−^ macrophages transfected with CD36 or control (Ctr) siRNA. (**G**,**H**) NFκB activation and (**F**). Cytokine secretion in bone marrow–derived macrophages from wild-type mice transfected with CD36 or control (Ctr) plasmids. NFκB activation was detected by immunoblotting using antibodies against phosphorylated p65 subunit of NFκB (pNFκB). Membranes were stripped and incubated with antibodies against total p65 subunit of NFκB (NFκB). The images are the crops of the full length blots. The full length blots are presented in Supplementary Fig. S9 and Supplementary Fig. S10. The secreted cytokines were detected in the cell supernatants. Interleukin 10 (IL-10), interferon gamma (INFγ), interleukin 12 p70 (IL-12), interleukin 6 (IL-6), tumor necrosis factor alpha (TNF-α), chemokine (C-X-C motif) ligand 1 (CXCL1) and Regulated on Activation, Normal T Cell Expressed and Secreted (RANTES). Results are shown as mean ± SD *n* = 3−8 *p < 0.05, **p < 0.01 ***p < 0.001.

Increased glucose uptake and metabolism through GLUT1 has been shown to induce inflammation in macrophages^28^. In line, the global gene expression analysis indicated increased NFκB transcription factor activity in *Vim*^−/−^ macrophages (Supplementary Fig. S1). We therefore investigated the inflammatory response of bone marrow–derived macrophages from *Vim*^−/−^ and wild-type mice and confirmed higher activity of NFκB in *Vim*^−/−^ macrophages than in wild-type macrophages (Fig. 3A,B). We also showed significantly higher secretion of proinflammatory cytokines in *Vim*^−/−^ macrophages (Fig. 3C).

Since *Vim*^−/−^ macrophages displayed increased surface expression of CD36, we tested if the proinflammatory response was, at least partly, mediated by CD36^25^. Results showed that knockdown of CD36 with siRNA in *Vim*^−/−^ macrophages decreased NFκB activation (Fig. 3D,E) and the secretion of proinflammatory cytokines (Fig. 3F), whereas overexpression of CD36 in wild-type macrophages induced increased NFκB activation (Fig. 3G,H) and increased secretion of proinflammatory cytokines (Fig. 3I). The CD36 protein expression levels after CD36 knockdown and CD36 overexpression are shown in Supplementary Fig. S6. These results indicate that vimentin deficiency in macrophages *in vitro* is linked to CD36-mediated inflammation.

### Decreased Atherogenesis Despite Increased Vascular Inflammation and Increased CD36 expression on macrophages in *Vim*^−/−^ Mice

To investigate the role of vimentin deficiency in macrophages *in vivo*, lethally irradiated low-density lipoprotein receptor–deficient (*Ldlr*^−/−^) mice were transplanted with bone marrow cells from vimentin-deficient (*Vim*^−/−^) mice or wild-type littermates and fed an atherogenic diet for 15 weeks. Body weight and plasma lipids did not differ between the two groups (Supplementary Tables S1 and S2).

**Figure 4:**
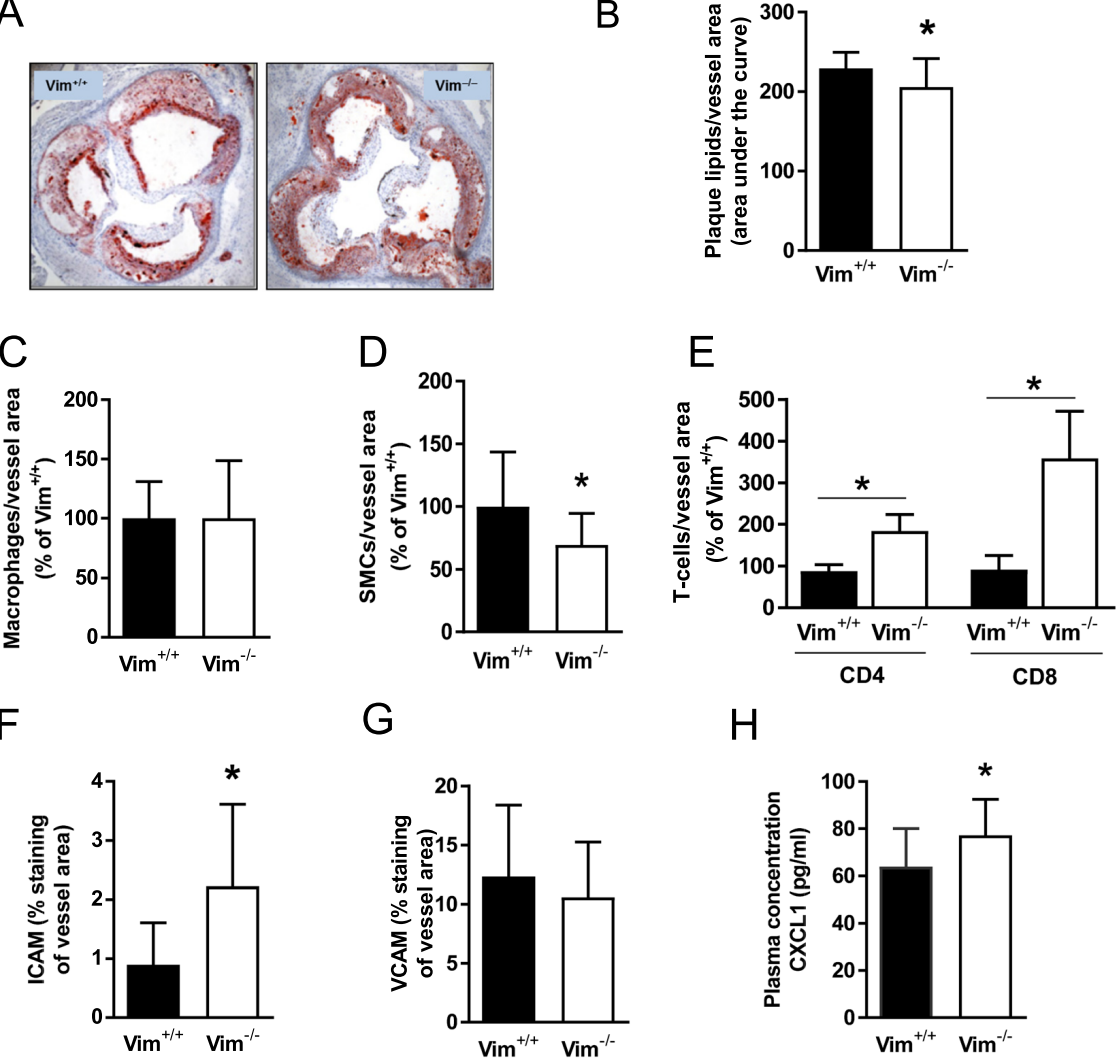
Reduced sub-endothelial lipid accumulation despite increased inflammation in aortas of *Ldlr*^−/−^ mice transplanted with bone marrow derived from *Vim*^−/−^ mice. (**A**) Representative images of Oil Red O staining of the aortic root of *Ldlr*^−/−^ mice transplanted with *Vim*^−/−^ or wild-type *(Vim*^+/+^) bone marrow followed by 15 weeks of Western atherogenic diet (**B**). Quantification of lipid accumulation (Oil Red O) in the aortic root (area under curve at 200, 400 and 600 µm distance from the three aortic valve cups) of *Ldlr*^−/−^ mice transplanted with *Vim*^−/−^ or wild-type *(Vim*^+/+^) bone marrow. Area under the curve was calculated as described in material and methods. (**C**–**H**) Analysis of cell composition and inflammation in the aortic root of *Ldlr*^−/−^ mice transplanted with *Vim*^−/−^ or wild-type *(Vim*^+/+^) bone marrow: sections were stained with antibodies against (**C**). Mac-2 (macrophages), (**D**) α-actin (smooth muscle cells), (**E**) CD4/CD8 (T cells), (**F**) ICAM-1 and (**G**) VCAM-1. (**H**) Circulating chemokine CXCL1 in plasma of *Ldlr*^−/−^ mice transplanted with bone marrow from *Vim*^−/−^ or wild-type mice. Results are shown as mean ± SD, *n* = 16, *p < 0.05.

Results showed a small but significant decrease in subendothelial lipid accumulation in the aortic root of *Vim*^−/−^ mice (Fig. 4A,B). Immunohistochemical analysis showed similar numbers of macrophages in the aortic root in both groups of mice (Fig. 4C). In contrast, the number of CD4+ and CD8+ T cells was increased (Fig. 4D) and the number of smooth muscle cells was significantly reduced (Fig. 4E) in mice that received *Vim*^−/−^ bone marrow, indicating a more inflammatory phenotype. The NFκB target gene intercellular adhesion molecule 1 (ICAM-1), but not vascular cell adhesion molecule 1 (VCAM-1), was increased significantly in lesions of mice that received *Vim*^−/−^ bone marrow (Fig. 4F,G). In addition, the T cell–recruiting chemokine CXCL1 was increased in plasma from mice that received *Vim*^−/−^ bone marrow (Fig. 4H), indicating an effect of *Vim* deficiency on systemic inflammation.

**Figure 5:**
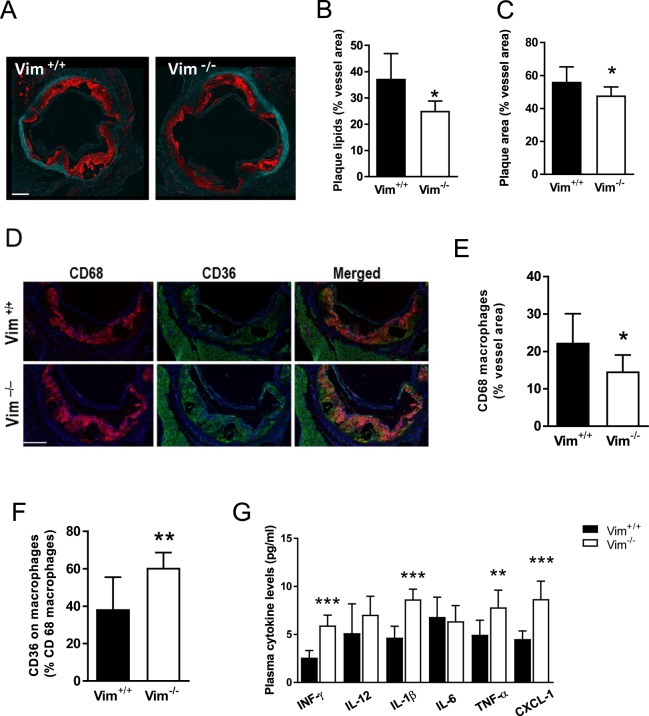
Reduced sub-endothelial lipid accumulation despite increased inflammation in *Vim*^−/−^ mice with atherosclerosis induced by injection with AAV8 virus containing PCSK9 gain of function mutant followed by 12 weeks of Western atherogenic diet. (**A**) Representative images of Oil Red O staining (in red) of the aortic root. Elastin layer is shown in green. Scale-bar represents 200 μm. (**B**) Quantification of lipid accumulation (Oil Red O) in the aortic root. (**C**) Quantification of plaque area in the aortic root. (**D**) Representative images of CD68 macrophage (red), CD36 staining (green) and CD36 on macrophages (CD68/CD36 merge) in the aortic root. Scale-bar represents 200 µm. (**E**) Quantification of CD68 positive macrophages in the aortic root. (**F**) Quantification of CD36 on CD68 positive macrophages in the aortic root. (**G**) Plasma proinflammatory cytokine profile of *Vim*^−/−^ and wild-type (*Vim*^+/+^) mice. Results are shown as mean ± SD, *n* = 6 *(Vim*^+/+^) and *n* = 8 *(Vim*^−/−^). *p < 0.05; **p < 0.01; ***p < 0.001.

Irradiation of mice induces tissue damage and inflammation. In contrast, AAV viral infection does not elicit any tissue damage or immunologic response^30^. We therefore also injected *Vim*^−/−^ and wild-type littermates with a single injection of a gain-of-function PCSK9 virus to induce hypercholesterolemia^31^. Body weight and plasma lipids did not differ between the two groups (Supplementary Table S3). In line with the results from the bone-marrow transplantation experiment, the subendothelial lipid accumulation was significantly decreased in the aortic root of *Vim*^−/−^ mice (Fig. 5A–C). In addition, the plaque size and CD68 macrophages were also significantly decreased in the aortic root of *Vim*^−/−^ mice (Fig. 5E). Importantly, immunohistochemistry studies revealed increased expression of CD36 on macrophages in the aortic root of *Vim*^−/−^ mice (Fig. 5D,F). We also showed significantly higher plasma levels of the proinflammatory cytokines INF-γ, IL-1β, TNF-α, CXCL-1 (Fig. 5G) in *Vim*^−/−^ mice. These results support increased vascular and systemic inflammation in *Vim*^−/−^ mice.

As vimentin deficiency impair endothelial function^21,32,33^, we tested if the decreased subendothelial lipid accumulation in *Vim*^−/−^ mice was caused by compromised transcellular transport of LDL through the endothelium. Alexa fluor-594-labelled LDL was injected into *Vim*^−/−^ and control mice, and the fluorescent signal of the labelled LDL in sections of the aortic root was quantified six hrs after injection. However, no difference was detected between *Vim*^−/−^ mice and control mice, indicating that transport of LDL through the endothelium is not impaired in *Vim*^−/−^ mice (Supplementary Fig. S7).

Together, these data show that vimetin-deficient mice display modestly decreased atherogenesis despite marked vascular inflammation with increased CD36 expression on macrophages.

## Discussion

Here we elucidated the role of vimentin in atherogenesis. Analysis of bone marrow–derived macrophages from *Vim*^−/−^ mice showed impairment of extracellular lipoprotein uptake, increased markers of oxidative stress, increased GLUT1-mediated glucose uptake, increased CD36 expression, augmented secretion of proinflammatory cytokines, and activation of NFκB. We also linked CD36 to the inflammatory response and showed increased CD36 expression on macrophages in atherosclerotic lesions *in vivo*.

Atherogenesis is initiated by subendothelial accumulation of atherogenic lipoproteins^9^. The retained lipoproteins become modified (e.g., aggregated and oxidized), and elicit a series of biological responses that develop into an inflammatory response^8^. Thus, the vascular inflammation is caused by retained artery wall lipoproteins. Interestingly, we show that *Vim*^−/−^ mice developed significantly less atherosclerosis despite increased vascular inflammation.

To clarify the underlying molecular mechanisms we tested if the decreased atherogenesis in *Vim*^−/−^ mice was due to reduced transendothelial transport of LDL, as vimentin deficiency has been shown to impair endothelial function^21,32,33^. However, results indicated that transport of LDL through the endothelium was not impaired in *Vim*^−/−^ mice. We also tested if *Vim*^−/−^ macrophages displayed altered uptake of LDL, and showed reduced uptake of native LDL but increased uptake of oxLDL.

Macrophage uptake of native LDL is mediated by macropinocytosis, a receptor independent endocytosis^34^. Once internalized, the LDL is sorted to late endosomes and lysosomes where the cholesterol-esters are degraded to free cholesterol, re-esterified and stored in lipid droplets forming foam cells^35^. Vimentin interacts with Ras-related protein (Rab)-7a and Rab9^36–38^, two proteins that are important in the late endocytic pathway^39,40^. A lack of this interaction may explain the results from the global gene expression profiling indicating an impaired plasma membrane to endosome transport in *Vim*^−/−^ macrophages. Interestingly, oxLDL is taken up differently by macrophages than native LDL, as binding of oxLDL to CD36 leads to endocytosis through a mechanism that is distinct from macropinocytosis^41^. This difference in endocytosis, may explain why uptake of native LDL is selectively impaired in *Vim*^−/−^ macrophages.

The role of oxLDL in atherogenesis was recently elegantly demonstrated by Que X *et al*. who showed that antibodies recognizing the oxidized phosphocholine present on oxLDL, block the uptake of oxLDL to macrophages and greatly reduced atherosclerosis and inflammation^42^. Therefore, our results showing increased uptake of oxLDL in *Vim*^−/−^ macrophages, despite modestly reduced atherosclerosis may seem counterintuitive. The underlying molecular mechanisms for this remain unclear but may be linked to an impaired transport of cholesterol to the site of re-esterification in *Vim*^−/−^ macrophages^43^. It is also possible that the impaired uptake of native LDL in *Vim*^−/−^ macrophages may, at least partly, contribute to the reduced atherosclerosis *in vivo*, as uptake of native LDL via macropinocytosis may contribute to foam cell formation during atherogenesis^34,35^.

Our results confirmed that vimentin deficiency in macrophages leads to increased oxidative stress^13,16^. Interestingly, we also showed increased GLUT1-mediated glucose uptake, in line with earlier studies showing that ROS augments glucose uptake^26,27^. Glucose is a critical component in the proinflammatory response of macrophages^28^, and our results indicate that the proinflammatory response was, at least partly, mediated by CD36, a central regulator of inflammasome activation^44^.

The regulation of CD36 is complex as the expression of CD36 is regulated at the transcriptional level by various cellular stimuli, including the transcription factor Nrf2 (nuclear factor-erythroid 2-related factor 2)^32,34^. Under conditions of oxidative stress, Nrf2 is translocated to the nucleus where it initiates transcription of antioxidative genes including CD36^45^. CD36 expression has also been shown to be stimulated by metabolites of arachidonic acid through ROS production^46^, and a recent study showed that ROS mediates cholesterol crystals-induced CD36 expression and foam cell formation^47^. Thus, the increased production of ROS in *Vim*^−/−^ macrophages could potentially mediate increased expression of CD36 through several mechanisms. However, CD36 mRNA expression was not altered by vimentin deficiency, suggesting that posttranslational regulation might be most important in determining the increased CD36 protein levels observed in *Vim*^−/−^ macrophages. Notably, glucose augments surface expression of CD36 at the level of translation in macrophages^29^.

The role of CD36 in atherogenesis has been controversial^48^. Febbraio *et al*. crossed a *Cd36*-null strain with the atherogenic *ApoE*-null strain and found a 76% decrease in aortic tree lesion area (Western diet) when compared with controls^49^. Febbraio *et al*. also reported that mice transplanted with *Cd36*-null bone-marrow were profoundly protected against atherosclerosis, and that re-introduction of macrophages with CD36 induced a twofold increase in the atherosclerotic lesion area^50^. In line, recent studies support a major role for CD36 in atherosclerotic lesion development *in vivo*^44,48,51–54^. However, Moore *et al*. presented different results, *ApoE/Cd36* double-null mice that were fed a high-fat diet had a modest reduction only or even an increase in some atherosclerotic lesions compared with *ApoE*-null mice^55^. The reason for these conflicting results is unclear^56,57^.

In conclusion, vimentin is highly abundant in activated macrophages and foam cells but its role during atherogenesis has been unknown. Here we demonstrate that vimentin has a strong suppressive effect on ROS and that vimentin deficient mice display increased vascular inflammation with increased CD36 expression on macrophages despite decreased subendothelial lipid accumulation. These findings demonstrate that vimentin has a key role in regulating the inflammation in macrophages during atherogenesis.

## Methods

### Cell Culture

Bone marrow cells were isolated from *Vim*^−/−^ and wild-type littermate mice. Femur was cleaned from muscle and tissue and bone marrow was flushed out using DMEM containing 2% heat inactivated fetal calf serum (FCS) as described^58^. See extended Method section on-line. Isolated marrow was washed with PBS containing 10 mM EDTA, red blood cells were lysed in 2% acetic acid, washed once more in PBS EDTA and plated in high-glucose DMEM supplemented with 10% FCS, 1% HEPES, 1% glutamine, 1% gentamicin, 0.01% β-mercaptoethanol, and 10% whole supernatant of cell line CMG14-12 as a source of mouse M-CSF^58^. Experiments were performed on differentiated macrophages 7–10 days after plating. Experiments were performed at least twice with macrophages prepared on different days.

### Gene expression analysis

Total RNA was prepared as previously described^59^ from bone marrow–derived macrophages from *Vim*^−/−^ and wild-type littermate mice and gene expression was measured using the Affymetrix Mouse Genome 430 2.0 Array. Raw probe intensity values were background corrected, normalized with quantile normalization, transformed to the log2 scale, and summarized into probe sets using the Robust Multichip Analysis algorithm^60^. Pair-wise comparison of the gene expression in *Vim*^−/−^ and wild-type macrophages was performed using the Piano R package^61^ (Supplementary Fig. S8). Raw data have been deposited to the GEO database: http://www.ncbi.nlm.nih.gov/geo/query/acc.cgi?token=kbqfcegmvhyntkx&acc=GSE63653.

### mRNA Expression in Bone Marrow Macrophages

Total RNA was extracted using RNeasy Kit (QIAGEN) and cDNA was synthesized using the high capacity cDNA Reverse Transcription Kit (Applied Biosystems). mRNA expression of genes of interest was analyzed with TaqMan real-time PCR in an ABI Prism 7900 HT Detection System (Applied Biosystems).

### *In Vitro* Analysis of Lipoprotein Uptake and Lipid Accumulation

LDL was isolated from human plasma by sequential ultracentrifugation^62^. Mildly oxidized LDL (oxLDL) was prepared by oxidation of LDL with 5 µM CuSO4 for 8 h at 37 °C^63^. LDL and oxLDL were labelled with 1,1′-dioctadecyl-1-3,3,3′,3′-tetramethylindocarbocyanin (Dil)^64^. Macrophages were incubated in medium without serum with 10 µg/ml of Dil-labelled LDL or Dil-labelled oxLDL for 3 h before fixation. The incubation with Dil-labelled oxLDL was done in the presence of 180 µM oleic acid^65^. Micrographs were captured by fluorescence scanner microscopy and lipoprotein uptake was determined by measuring the intracellular fluorescent area per cell^66^. Bone marrow–derived macrophages were incubated for 16 h with 180 μM oleic acid. The total Oil Red O surface area was quantified as described^67^. Macrophage lipids were extracted^68^ and quantified using a combination of HPLC and mass spectrometry according to previous work^69^. The use of human plasma was approved by the Regional ethical review board in Gothenburg, informed consent was obtained for the use of plasma, and the study was performed conform the declaration of Helsinki.

### *In Vitro* Analysis of Macropinocytosis

Bone marrow–derived macrophages were incubated with dextran labelled with Alexa Fluor 488 (200 µg/ml) in the media for 16 h^59^. The fluorescence was assessed with a fluorimeter (MDS Analytical Technologies), and normalized to protein content.

### FACS Analysis

Bone marrow–derived macrophages were resuspended in FACS buffer (PBS, 3% FCS, 0.09% NaAz), incubated with Fc block (2.4G2, BD Bioscience) and then with antibodies directed against surface antigens [monoclonal rabbit anti-mouse LDLr (Abcam), monoclonal goat anti-mouse VLDLr antibody (R&D Systems) with secondary antibody APC-conjugated donkey anti-goat F(ab′)2 antibody (Santa Cruz Biotechnology), rat anti-mouse MSR1-FITC (Lifespan Biosciences), rabbit anti-mouse ABCG1 (Abcam), rat anti-mouse ABCA1–FITC (Novus Biologicals), rabbit anti-mouse SR-B1 (Novus Biologicals), and armenian-hamster anti-mouse CD36-APC (Abcam)].

After the initial surface-staining step, cells were fixed with paraformaldehyde, permeabilized in FACS buffer with 0.5% saponin and then stained for intracellular CD68 using rat-anti-mouse–PE antibodies (AbD Serotec). 10,000 cells were collected for each staining using a FACSCanto II equipped with the Diva 6:2 software (BD Bioscience) and were analyzed using the FlowJo software (Tree Star).

### Immunoblot

Immunoblot analysis was performed as described previously^66^ using antibodies against p-p65 NFκB and total p65 NFκB (Cell Signaling Technology 3033, 8242), CD36 (R&D Systems AF2519) and GLUT1 (Abcam 115730). Protein levels were normalized against β-actin (Abcam 8226).

### Glucose Uptake and Glucose/Mannose Treatment

For glucose uptake, bone marrow–derived macrophages were incubated for 10 min at 37 °C with 1 µCi/ml 2 deoxy-D-(1-^3^H) glucose (Amersham) and 10 µM deoxyglucose (Sigma) in uptake buffer (140 mM NaCl, 20 mM HEPES, 5 mM KCl, 2.5 mM MgSO_4_*7H_2_O, 1 mM CaCl_2_*2H_2_O, pH 7.4). Cells were washed 3 times with ice cold PBS and lysed with 500 µl NaOH (0.2 M). Radioactivity was assessed in 300 µl cell lysate with a β-counter (Perkin Elmer) and normalized to the protein content. For glucose treatment, cells differentiated in medium with 4.5 mg/ml glucose were incubated overnight in medium with 2.5 mg/ml glucose and thereafter in medium containing 6 mg/ml glucose^29^. Control cells received 6 mg/ml mannose instead.

### Transfection

For CD36 overexpression, isolated bone marrow–derived macrophages were transfected with CD36 or control plasmids using Lipofectamine 2000 (Invitrogen). For CD36 and GLUT1 knockdown, differentiated bone marrow–derived macrophages (2–2.5 × 10^5^ cells) were transfected after overnight plating with CD36, GLUT1 or scrambled siRNA (Applied Biosystems). Cells were harvested 48 h after transfection.

### *In Vitro* Analysis of Cytokine Secretion

Cytokine levels in the media were analyzed with a SECTOR Imager 2400 reader (Meso Scale Discovery).

### Analysis of TBARS and H_2_O_2_

Thiobarbituric acid-reactive substances (TBARS) were determined as described^70^. Fluorescence was measured at 553 nm with 515 nm excitation. Levels of hydrogen peroxide equivalents (H_2_O_2eq_) were analyzed in cultured bone marrow–derived macrophages^70^. The assay is based on the oxidation of ferrous ions to ferric ions by hydrogen peroxide at acidic pH (OXIS International).

### Lipid extraction, lipid class fractionation and lipid analysis using mass spectrometer

Lipids were analyzed according to previous work^71^. See extended Method section on-line.

### Mice

Vimentin-deficient (*Vim*^−/−^)^72^, wild-type littermate, and low-density lipoprotein receptor deficient (*Ldlr*^−/−^) mice (JAX^®^ Mice, Stock Number #002207) on C57BL/6J background were housed in a pathogen-free barrier facility and fed rodent chow. All mice were housed in a barrier facility, and experiments were conducted according to protocols approved by the Gothenburg Ethics Committee. All animal procedures were performed in line with the Directive 2010/63/EU of the European Parliament on the protection of animals used for scientific purposes. At the end of experiments, mice were sacrificed using isoflurane and cervical dislocation.

### Bone Marrow Transplantation

Bone marrow transplantation of 32 female 6-week-old *Ldlr*^−/−^ mice was performed as described^59,73^.

### Induction of Atherosclerosis in Mice using Virus-mediated Overexpression of Mutant PCSK9

Atherosclerosis was induced in female *Vim*^−/−^ or wild type littermates controls by intravenous injection of adeno-associated viruses containing gain of function mutant (D377Y) of mouse PCSK9 (1.5 × 10^11^ vector genomes/mouse)^31^. One day after virus injection of mouse PCSK9, mice were given western atherogenic diet for 12 weeks^31^. Plasma cholesterol and triglyceride levels were collected after 4 h fasting and analyzed using Infinity kits (Sigma).

### Analysis of Aortae

The aortic roots were embedded in OCT Tissue-Tec medium, frozen in dry ice and isopentane, cut into 10-µm-thick cross sections starting from the commissures of the aortic cups upwards, and stained with 0.5% Oil Red O^74^. For bone marrow transplantation model Oil Red O stained area in the plaque (plaque lipids) and vessel area (excluded the lumen) were quantified at 200, 400 and 600 µM distance from the aortic valve cups for each mouse using KS-400 software (Zeiss). The ratio of plaque lipids/vessel area was calculated for each level and thereafter area under the curve was calculated for each mouse. The results are presented as area under the curve for each experimental group. Immunohistochemistry was done with antibodies against α-actin, Mac-2, CD4/CD8, ICAM and VCAM^73^ on cross sections of aortic sinuses. For PCSK9-induced model of atherosclerosis sections at 200–240 µm distance from the three aortic valve cups were stained with 0.5% Oil Red O. Images were acquired using Metasystem automated slide scanner (MetaSystems, Germany) equipped with SpectraSplit^TM^ filter system for extended multicolour imaging and a Carl Zeiss AxioImager.Z2 microscope. The Oil Red O fluorescent signal was detected using the Texas red filter (excitation BP 530–585; emission LP 615)^75^. The Oil Red O stained area in the plaque (plaque lipids) and vessel area (excluded the lumen) were quantified using Visiopharm software program version 5.3.0.1562 (Denmark). The lipid accumulation in the plaque was estimated as percent of vessel area.

### Analysis of CD36 on Macrophages in Atherosclerotic Lesions

At sacrifice, the aortic roots were embedded in OCT Tissue-Tec medium, frozen in dry ice and isopentane, sectioned consecutively (10 µM sections) starting from the commissures of the aortic cups upwards. Sections at 200–240 µm distance from the three aortic valve cups were fixed with 2% formaldehyde, blocked with 1% BSA and Fc block (BD Bioscience), and stained with rat anti-CD68-Alexa fluor 594 antibodies (Biolegend) and goat anti-CD36 (R&D systems) antibodies followed by donkey anti-goat Alexa fluor 488 conjugated secondary antibodies. Images were acquired using Metasystem automated slide scanner (MetaSystems, Germany) equipped with SpectraSplit TM filter system for extended multicolour imaging and a Carl Zeiss AxioImager.Z2 microscope. The total macrophage area and the area of CD36 positive macrophages in the atherosclerotic lesions were quantified using Visiopharm software program version 5.3.0.1562 (Denmark). The CD36 positive macrophages were estimated as percentage of total macrophage area.

### Blood Analysis

Blood was obtained after a 4 h fast the day before the mice were sacrificed. Cholesterol and triglycerides were measured on a Konelab 20 autoanalyzer (Thermo, Vantaa, Finland). Plasma cytokines were analyzed with a SECTOR Imager 2400 reader (Meso Scale Discovery, Gaithersburg, MD).

### Statistical analysis

Data are shown as means ± SD. Measurements were compared with the two-tailed *t*-test, Mann-Whitney rank sum test or one-way ANOVA with Sidak’s or Dunnett’s multiple comparison tests.

## Electronic supplementary material

Supplementary data

## Data Availability

The datasets generated during and/or analyzed during the current study are available from the corresponding author on reasonable request.
