# Supplementary material for: Vimentin deficiency in macrophages induces increased oxidative stress and vascular inflammation but attenuates atherosclerosis in mice
Source: Sci Rep. 2018 Nov 19;8:16973. doi: 10.1038/s41598-018-34659-2 (PMC6242955; doi:10.1038/s41598-018-34659-2)
Supplement: Supplementary file 1 — Supplementary data [file 41598_2018_34659_MOESM1_ESM.pdf]

Supplementary Methods, Tables and Figures for *"Vimentin deficiency in macrophages induces increased oxidative stress and vascular inflammation but attenuates atherosclerosis in mice"* by Håversen *et al.*

SUPPLEMENTARY INFORMATION

Vimentin deficiency in macrophages induces increased oxidative stress and vascular inflammation but attenuates atherosclerosis in mice

Liliana Håversen[1], Jeanna Perman Sundelin[2], Adil Mardinoglu[3], Mikael Rutberg[1], Marcus Ståhlman[1], Ulrika Wilhelmsson[4], Lillemor Mattsson Hultén[1], Milos Pekny[4], Per Fogelstrand[1], Jacob Fog Bentzon[5], Malin Levin[1], and Jan Borén[1*]

[1]Department of Molecular and Clinical Medicine/Wallenberg Laboratory, University of Gothenburg, and Sahlgrenska University Hospital, Gothenburg, Sweden

[2]Strategic planning and operations, Cardiovascular and metabolic diseases, IMED Biotech Unit, AstraZeneca Gothenburg Sweden

[3]Systems and Synthetic Biology Group, Chalmers University of Technology, Gothenburg, Sweden

[4]Department of Clinical Neuroscience/Center for Brain Repair, University of Gothenburg, Gothenburg, Sweden

[5]Department of Clinical Medicine, Aarhus University, Aarhus, Denmark, and Centro Nacional de Investigaciones Cardiovasculares Carlos III (CNIC), Madrid, Spain

Correspondence and requests for materials should be addressed to J.B.
(email: jan.boren@wlab.gu.se)

Supplementary Methods, Tables and Figures for *"Vimentin deficiency in macrophages induces increased oxidative stress and vascular inflammation but attenuates atherosclerosis in mice"* by Håversen *et al.*

METHODS

Cell Culture**—**Bone marrow cells were isolated from $Vim^{-/-}$ and wild-type littermate mice. Femur was cleaned from muscle and tissue and bone marrow was flushed out using DMEM containing 2% heat inactivated fetal calf serum (FCS). Isolated marrow was washed with PBS containing 10 mM EDTA, red blood cells were lysed in 2% acetic acid, washed once more in PBS EDTA and plated in high-glucose DMEM supplemented with 10% FCS, 1% HEPES, 1% glutamine, 1% gentamicin, 0.01% β-mercaptoethanol, and 10% whole supernatant of cell line CMG14-12 as a source of mouse M-CSF.[1] Experiments were performed on differentiated macrophages 7–10 days after plating. Experiments were performed at least twice with macrophages prepared on different days.

HPLC quantification and lipid class fractionation**—** Lipids were extracted according to Folch procedure.[2] Total lipid extracts were reconstituted in heptane-isopropanol (9:1; v/v) and separated using normal-phase HPLC according to previous work [3]. Using a post-column split, 75% of the HPLC flow was diverted to a Gilson FC 204 fraction collector (Gilson, Middleton, WI), which was used for fractionation of lipid classes for further analysis using mass spectrometry. The other 25% of the flow was diverted into an evaporative light scattering detector (Polymer Laboratories, Amherst, MA). This detector was used for quantification of CE, TAG and FC.

Lipid analysis using mass spectrometry**—**Lipids in total lipid extracts or in HPLC-isolated fractions were analyzed with precursor ion scanning in positive and negative ion modes using a QSTAR XL QqTOF mass spectrometer (MDS Sciex, Concord, Canada) equipped with a robotic nanoflow ion source, the TriVersa NanoMate (Advion BioSciences, Ithaca, NJ).[4, 5] Quantification and profiling of phosphatidylcholine (PC), ether-linked phosphatidylcholine (PC-O), sphingomyelin (SM) and lysophosphatidylcholine (LPC) were made directly from the total lipid extract. Profiling of CE and TAG was performed using the corresponding HPLC fractions. Diacylglycerol (DAG) was quantified and profiled from the HPLC fraction. Prior to analysis, HPLC fractions were evaporated and redissolved in chloroform-methanol (1:2 v/v) containing 5 mM ammonium acetate. Ceramides (CER) isolated by HPLC fractionation were quantified using reversed phase HPLC coupled to a triple quadrupole Quattro Premier mass spectrometer (Waters, Milford, USA) according to previous work.[6]

LDL-594 transport thought endothelium —Human LDL was conjugated with ATTO-594 NHS-ester (ATTO-TEK, Germany, AD 594-31) according to manufacturer´s recommendation. Mice were administrated the 594-labeled LDL (500 µg) through retro-orbital injection. The mice were sacrificed 6 h after injection. The aortic roots were embedded in OCT Tissue-Tec medium, frozen in dry ice and isopentane, sectioned consecutively (8 µM sections) starting from the commissures of the aortic cups upwards. Sections at 200, 400 and 600 µm distance from the three aortic valve cups were fixed with 2% formaldehyde, nuclei were stained with DAPI (2 µg/ml, 4 min) and the slides were mounted with ProlongGold. Images were acquired using Metasystem automated slide scanner (MetaSystems, Germany) equipped with SpectraSplit TM filter system for extended multicolor imaging and a Carl Zeiss AxioImager.Z2 microscope. The area of LDL-594 signal present in the arterial wall was quantified using Visiopharm software program version 5.3.0.1562 (Denmark).

REFERENCES

1. Takeshita S, Kaji K, Kudo A. Identification and characterization of the new osteoclast progenitor with macrophage phenotypes being able to differentiate into mature osteoclasts. *J Bone Miner Res* 2000;**15**:1477-1488.
2. Folch J, Lees M, Sloane Stanley GH. A simple method for the isolation and purification of total lipides from animal tissues. *J Biol Chem* 1957;**226**:497-509.
3. Homan R, Anderson MK. Rapid separation and quantitation of combined neutral and polar lipid classes by high-performance liquid chromatography and evaporative light-scattering mass detection. *J Chromatogr B Biomed Sci Appl.* 1998;**708**:21-26.
4. Ejsing CS, Moehring T, Bahr U, Duchoslav E, Karas M, Simons K, Shevchenko A. Collision-induced dissociation pathways of yeast sphingolipids and their molecular profiling in total lipid extracts: a study by quadrupole TOF and linear ion trap-orbitrap mass spectrometry. *J Mass Spectrom* 2006;**41**:372-389.
5. Ekroos K, Ejsing CS, Bahr U, Karas M, Simons K, Shevchenko A. Charting molecular composition of phosphatidylcholines by fatty acid scanning and ion trap MS3 fragmentation. *J Lipid Res* 2003;**44**:2181-2192.
6. Stahlman M, Pham HT, Adiels M, Mitchell TW, Blanksby SJ, Fagerberg B, Ekroos K, Boren J. Clinical dyslipidaemia is associated with changes in the lipid composition and inflammatory properties of apolipoprotein-B-containing lipoproteins from women with type 2 diabetes. *Diabetologia* 2012;**55**:1156-1166.

SUPPLEMENTARY TABLES

Supplementary Table S1. Characteristics of *Ldlr*$^{-/-}$ mice after bone marrow transplantation with *Vim*$^{-/-}$ or wild type (*Vim*$^{+/+}$) bone marrow followed by 15 weeks of Western atherogenic diet. *n* =16 (*Vim*$^{+/+}$), *n* =16 (*Vim*$^{-/-}$). S=serum.

	Vim$^{+/+}$	***Vim***$^{-/-}$	***P*-value**
Body Weight (g)	27.1±3.8	25.4±3.3	*ns*
S-cholesterol (mmol/L)	22.1±3.1	20.9±2.8	*ns*
S-triglycerides (mmol/L)	3.2±0.6	2.9±0.9	*ns*
S-free fatty acids (mmol/L)	0.69±0.19	0.72±0.31	*ns*

Supplementary Table S2. Relative quantification of lipids in *Vim*$^{+/+}$ and *Vim*$^{-/-}$ bone marrow–derived macrophages. Ratio of lipids (*Vim*$^{+/+}$/*Vim*$^{-/-}$) in bone marrow–derived macrophages is shown.

**Lipid Class**	***Vim***$^{+/+}$ ***vs. Vim***$^{-/-}$	***P* value**
Triglycerides	1.11±0.68	*ns*
Diglycerides	0.95±0.18	*ns*
Free Cholesterol	1.02±0.03	*ns*
Cholesteryl Ester	0.98±0.61	*ns*
Phosphatidylethanolamine	0.98±0.04	*ns*
Ether-Linked Phosphatidylcholine	0.97±0.08	*ns*
Phosphatidylcholine	1.03±0.11	*ns*
Lysophosphatidylcholine	0.98±0.34	*ns*
Sphingomyelin	1.03±0.14	*ns*
Ceramide	1.23±0.22	*ns*

Supplementary Table S3. Characteristics of *Vim*$^{-/-}$ and wild-type (*Vim*$^{+/+}$) mice after induction of atherosclerosis with AAV8 virus containing mouse PCSK9 gain of function mutant followed by 12 weeks of Western atherogenic diet. Results are shown as mean ± SD, *n* = 9 (*Vim*$^{+/+}$), n=9 (*Vim*$^{-/-}$). P=plasma

	Vim$^{+/+}$	***Vim***$^{-/-}$	***P*-value**
Body Weight (g)	32.1±2.2	34.3±4.2	*ns*
P-cholesterol (mmol/L)	32.6±4.9	33.5±5.2	*ns*
P-triglycerides (mmol/L)	4.1±1.0	4.8±1.0	*ns*

SUPPLEMENTARY FIGURES

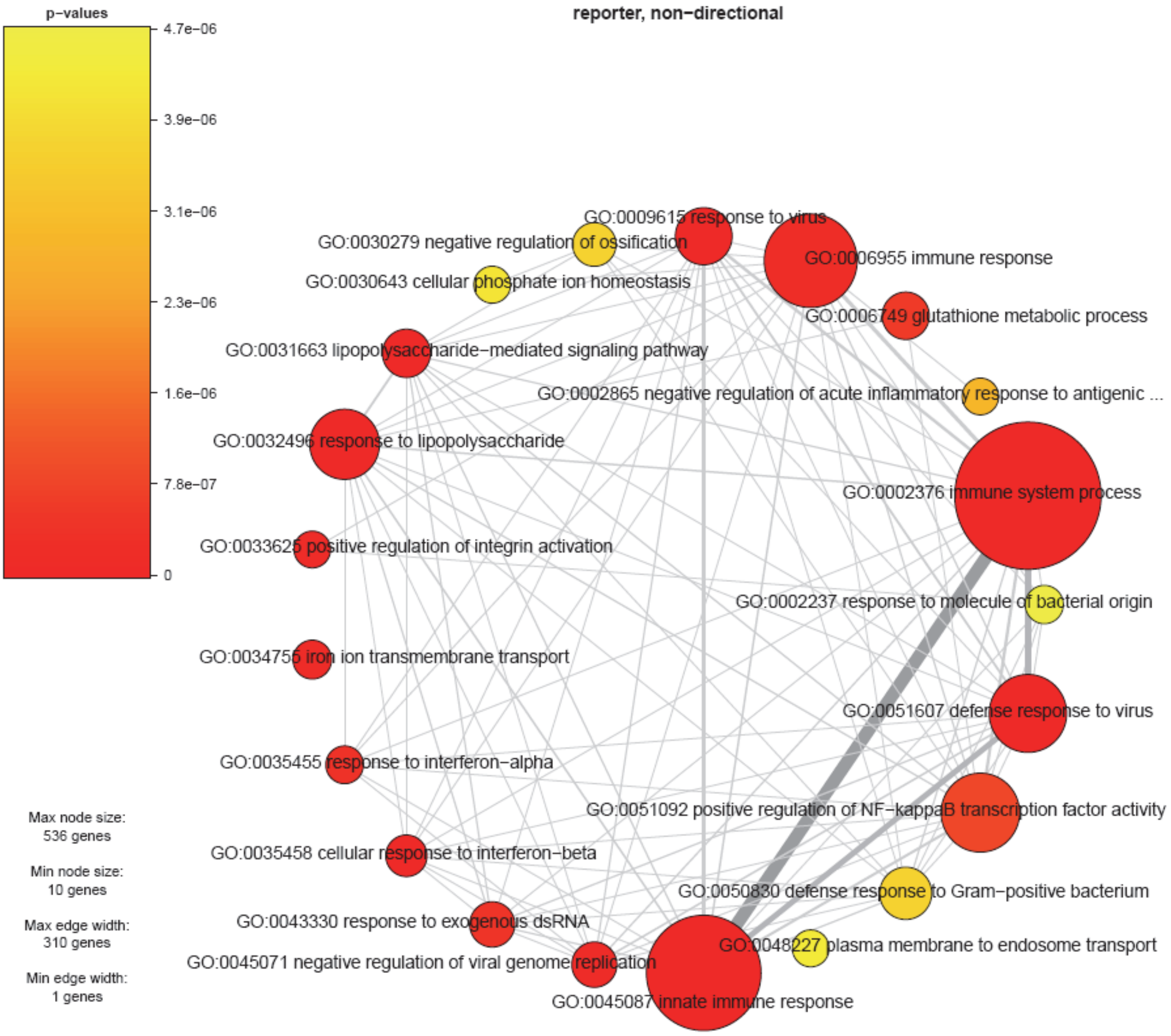

Supplementary Figure S1. Network plot for gene expression data from *Vim*$^{-/-}$ and wild-type macrophages showing the relation between significant gene sets (adjusted p<0.000005) for Gene Ontology biological process terms by connecting those that share similar genes. The thicknesses of the connecting lines correspond to the number of shared genes and the size of the nodes corresponds to the size of the gene sets. −Log (p values) are used for color coding.

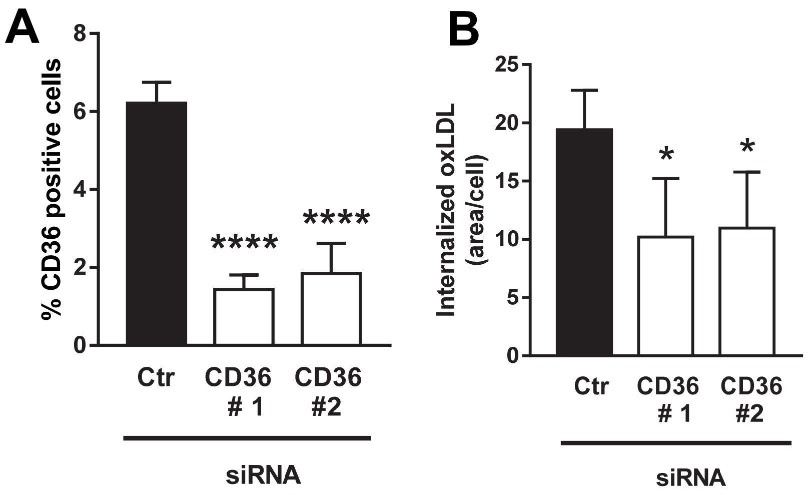

Supplementary Figure S2. Internalization of oxLDL is mediated via CD36 receptor in wild type bone marrow macrophages. **A**. CD36 positive cells as stained by immunohistochemistry with antibodies against CD36 after 48 h of knockdown with control and CD36 siRNA **B.** Internalization of dil oxLDL after 48 h of knockdown with control and CD36 siRNA. Data are mean ± SD; * $p < 0.05$; ****$p < 0.0001$, One –way ANOVA followed by Dunnett´s multiple comparison test, $n = 4$.

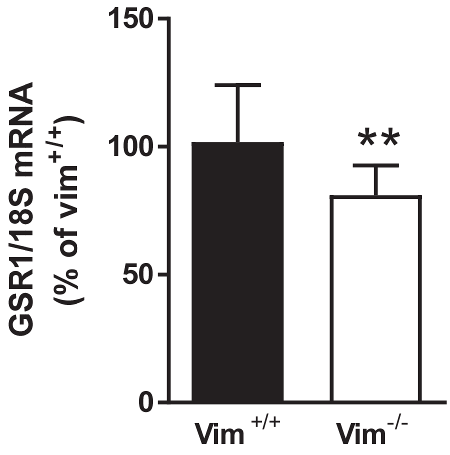

Supplementary Figure S3. Glutathione reductase (GSR1) mRNA expression (normalized to 18S) in bone marrow-derived macrophages isolated from *Vim*$^{-/-}$ and *Vim*$^{+/+}$ mice. Data are mean ± SD; ***p*$<$ 0.01, unpaired two-tailed t-test, *n* = 13 (*Vim*$^{+/+}$) and *n* =14 (*Vim*$^{-/-}$).

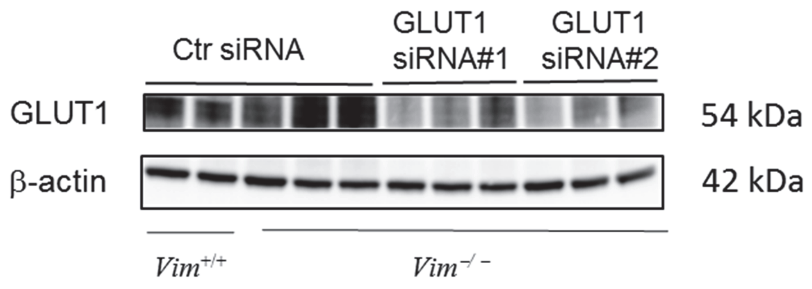

Supplementary Figure S4. Representative immunoblotting of GLUT1 protein expression in *Vim*$^{+/+}$ and *Vim*$^{-/-}$ macrophages after knockdown with control (Ctr) or GLUT1 siRNA. Two separated siRNA were used to knock-down GLUT1 in *Vim*$^{-/-}$ macrophages.

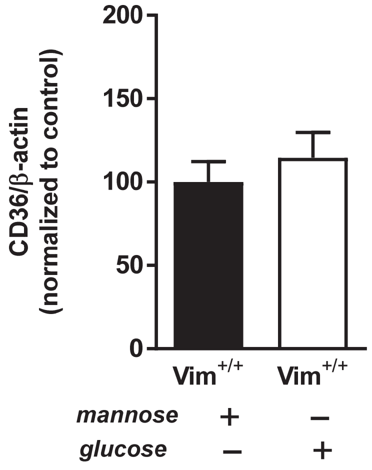

Supplementary Figure S5. CD36 protein levels of bone marrow-derived *Vim*$^{+/+}$ macrophages treated with 6 mg/ml glucose or mannose. Results are shown as mean ± SD, *n*=3.

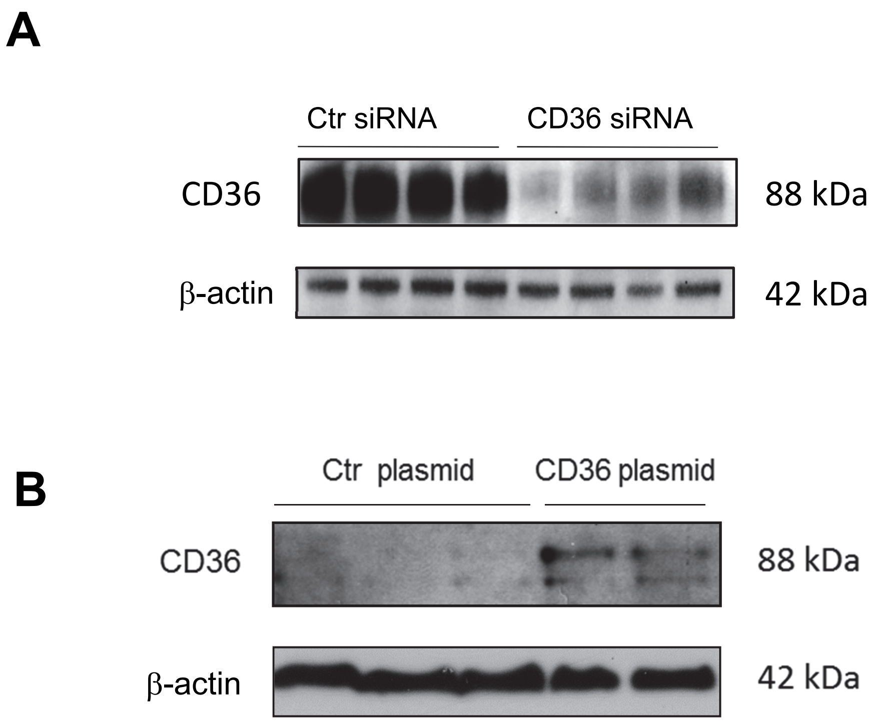

Supplementary Figure S6. A. Immunoblotting of CD36 protein expression after CD36 knockdown in *Vim*$^{-/-}$ bone marrow derived macrophages with siRNA. **B.** Immunoblotting of CD36 protein expression after CD36 overexpression in *Vim*$^{+/+}$ bone marrow derived macrophages with CD36 or control plasmids.

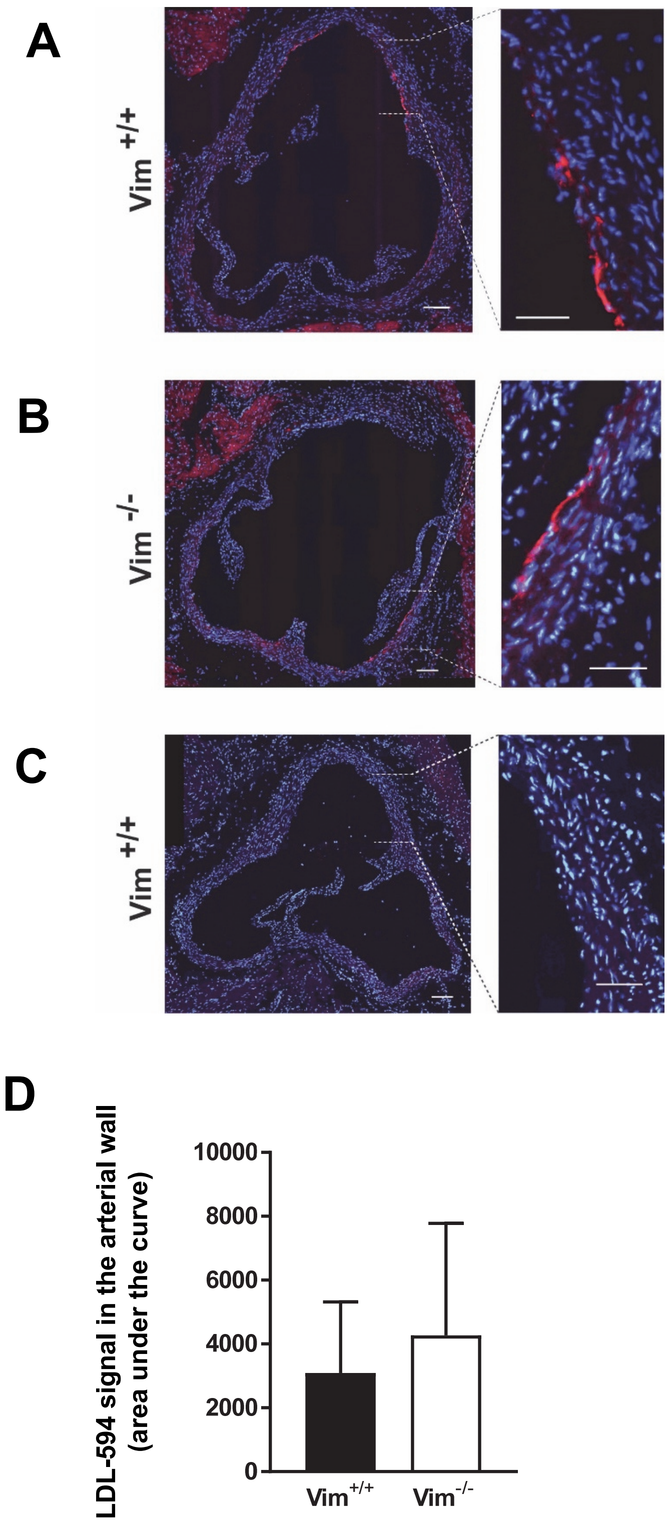

Supplementary Figure S7. The transendothelial transport of LDL into the arterial wall in *Vim*$^{-/-}$ and *Vim*$^{+/+}$ mice. Mice were injected with either human LDL labeled with Alexa fluor-594 fluorochrome (A and B), or with PBS for 6 h (C). The aortic roots were sectioned and the fluorescent LDL-594 was visualized by fluorescence microscopy (A–C); Red signal LDL-594, blue signal DAPI (nuclei); Scale-bar represents 50 µm. D. Quantification of LDL-594 signal in the arterial wall was performed as described in *Material and Methods* at 200, 400 and 600 µm distance from the three aortic valve cups, and area under the curve was calculated. Data are mean ± SD; n = 5 (*Vim*$^{+/+}$) and n=3 (*Vim*$^{-/-}$).

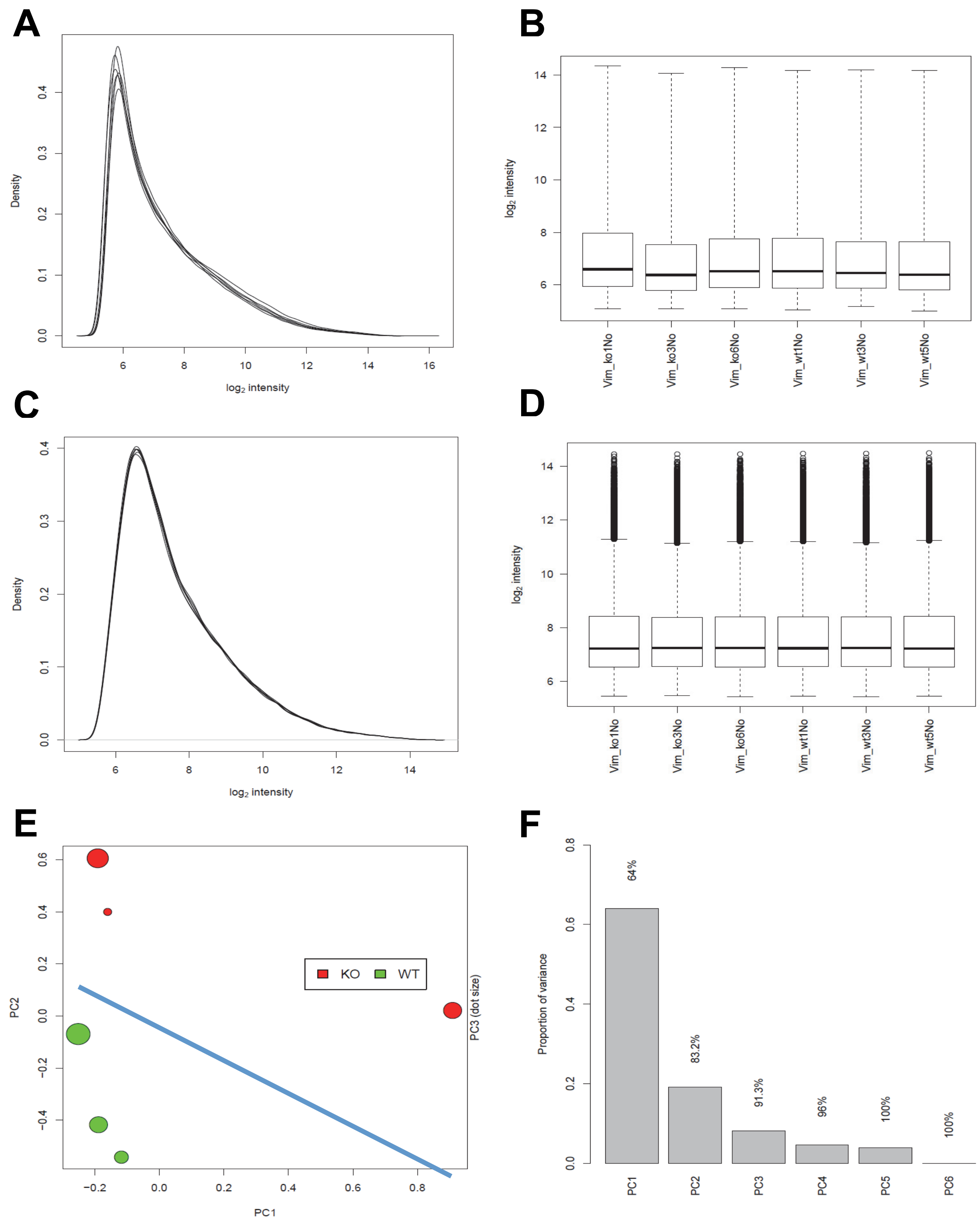

Supplementary Figure S8. Gene expression data from *Vim*$^{-/-}$ and wild-type macrophages were analyzed. Raw probe intensity values were background corrected, normalized with quantile normalization, transformed to the log2 scale, and summarized into probe sets using the Robust Multichip Analysis algorithm. **A.** Histogram and **B**. box plot of raw data. **C**. Histogram and **D**. box plot of normalized data. **E**. Principal component analysis of the data. **F**. Principal component importance.

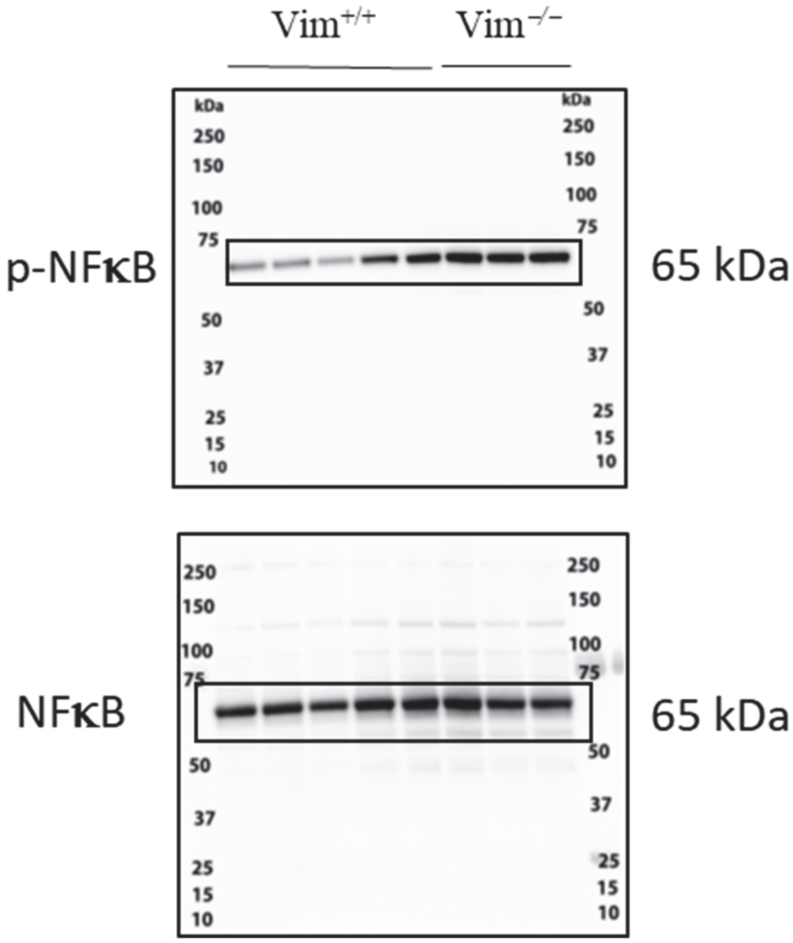

Supplementary Figure S9. Full-length blots for **Fig. 3A.** The membrane was first incubated with pNF-κB p65 antibodies and then stripped and incubated with NF-κB p65 antibodies. Images were acquired with ChemiDoc^{TM} Touch imaging system using optimal auto-exposure time for each antibody. The images presented in **Fig. 3A** are crops of the blot regions indicated by the black surrounding lines.

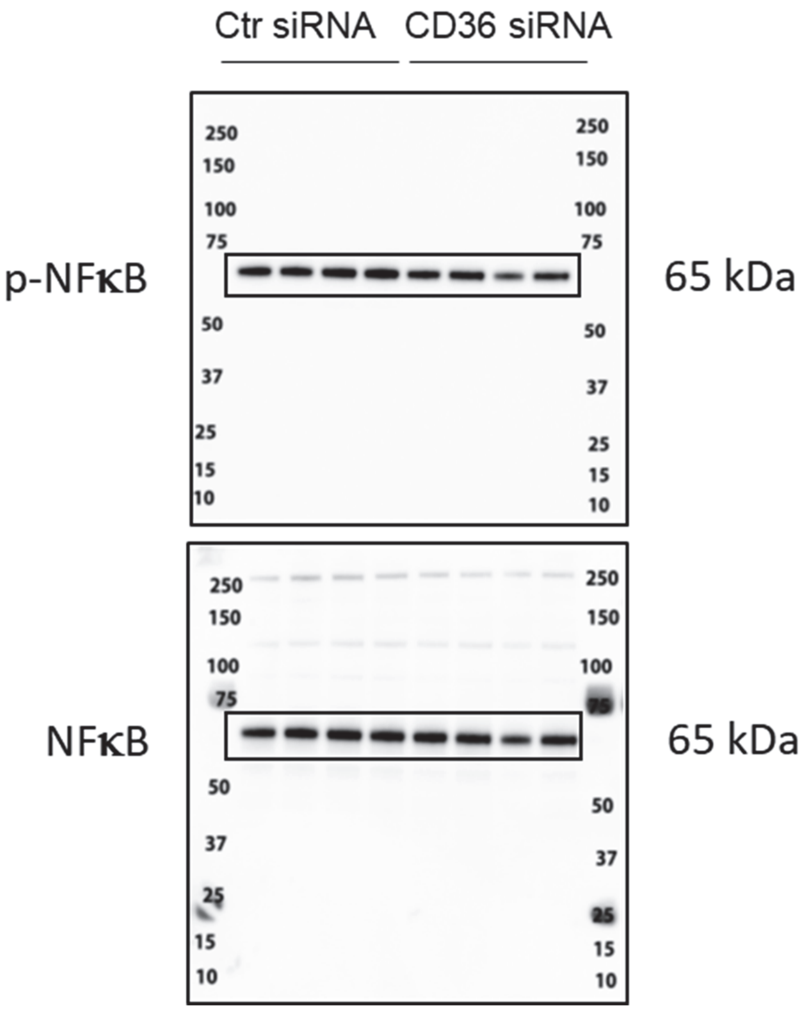

Supplementary Figure S10. Full-length blots for **Fig. 3D.** The membrane was first incubated with pNF-κB p65 antibodies and then stripped and incubated with NF-κB p65 antibodies. Images were acquired with ChemiDoc^{TM} Touch imaging system using optimal auto-exposure time for each antibody. The images presented in **Fig. 3D** are crops of the blot regions indicated by the black surrounding lines.
